# Evaluating the Patterns of FAPI Uptake in the Shoulder Joint: a Preliminary Study Comparing with FDG Uptake in Oncological Studies

**DOI:** 10.1007/s11307-023-01893-8

**Published:** 2024-01-04

**Authors:** Yohji Matsusaka, Rudolf A. Werner, Sebastian E. Serfling, Andreas K. Buck, Aleksander Kosmala, Takanori Sasaki, Alexander Weich, Takahiro Higuchi

**Affiliations:** 1 Department of Nuclear Medicine and Comprehensive Heart Failure Center (CHFC), Molecular Imaging of the Heart, University Hospital of Würzburg, Oberdürrbacher Str. 6, ZIM House A4, 97080 Würzburg, Germany; 2 Division of Nuclear Medicine and Molecular Imaging, The Russell H Morgan Department of Radiology and Radiological Sciences, Johns Hopkins School of Medicine, Baltimore, MD USA; 3 Faculty of Medicine, Dentistry and Pharmaceutical Sciences, Okayama University, Okayama, Japan; 4 Internal Medicine II and ENETS CoE NET-Zentrum Würzburg, Gastroenterology, University Hospital Würzburg, Würzburg, Germany

**Keywords:** Fibroblast activation inhibitor, Shoulder, Acromioclavicular joints, F-18 fluorodeoxyglucose, Positron emission tomography, FAP, Ga-68 FAPI-04, Rheumatoid arthritis, Osteoarthritis

## Abstract

**Background:**

Fibroblast activation protein inhibitor (FAPI) targeting PET has been introduced as a novel molecular imaging modality for visualizing cancer-associated fibroblasts. There have also been reports suggesting incidental findings of localized accumulation in the shoulder joints. However, further characterization in a larger patient cohort is still lacking.

**Methods:**

77 consecutive patients (28 females; mean age, 63.1 ± 11.6) who underwent Ga-68 FAPI-04 PET/CT for diagnosis of solid tumors were included. The incidence and localization of tracer uptake in shoulder joints were investigated and compared with available F-18 FDG scans serving as reference.

**Results:**

Ga-68 FAPI-04 uptake was evaluated in 77 patients (154 shoulder joints), of whom 54 subjects (108 shoulder joints) also had available F-18 FDG scans for head-to-head comparison. On FAPI-targeted imaging, 67/154 shoulders (43.5%) demonstrated increased radiotracer accumulation in target lesions, which were distributed as follows: acromioclavicular (AC) joints in 25/67 (37.3%), followed by glenohumeral and subacromial (GH + SA) joints in 23/67 (34.3%), or both (AC and GH + SA joints) in the remaining 19/67 (28.4%). Ga-68 FAPI-04 correlated with quantified F-18 FDG uptake (r = 0.69, *p* < 0.0001). Relative to the latter radiotracer, however, *in-vivo* FAP expression in the shoulders was significantly increased (Ga-68 FAPI-04, 4.7 ± 3.2 vs F-18 FDG, 3.6 ± 1.3, *p* < 0.001).

**Conclusion:**

Our study revealed focal accumulation of Ga-68 FAPI-04 in the shoulders, particularly in the AC joints, with higher uptake compared to the inflammatory-directed PET radiotracer F-18 FDG in oncological studies. As a result, further trials are warranted to investigate the potential of FAPI-directed molecular imaging in identifying chronic remodeling in shoulder joints. This could have implications for initiating anti-FAP targeted photodynamic therapy based on PET signal strength.

## Introduction

Persistent shoulder pain, including frozen shoulder, is one of the most frequent complaints across all generations, with a prevalence of 26% and a lifetime estimate of 67% [1]. Among others, chronic inflammation along with increased fibroblast proliferation and extracellular matrix deposition have been advocated to contribute to disease on-set and progression, as recently demonstrated in patients with idiopathic frozen shoulder undergoing surgery [2]. Moreover, also classified as chronic inflammatory diseases, shoulder-associated rheumatoid (RA) and osteoarthritis (OA) are also characterized by increased fibroblast activation protein (FAP) expression in chondrocytes, in particular in the superficial zone of cartilage tissues, thereby rendering FAP as an attractive, pro-inflammatory target [3, 4]. Pathologically, fibrosis by fibroblasts causes extracellular matrix stiffness, which can lead to contracture, ultimately limiting motion of joints [5]. Contractures, however, reduce the patient's quality of life (QOL). If the degree of joint fibrosis can be determined by non-invasive imaging modalities, it may be possible to delay the respective disease progression, preferably for initiating treatment at the right time for the right patient. Computed tomography (CT), magnetic resonance image (MRI), and ultrasonography are clinically used as indices of joint morphologic evaluation. While they can visualize lesions on a millimeter scale [6–8], it is difficult to assess the activity at the cellular and molecular level within the lesion.

In recent years, fibroblast activation protein inhibitor (FAPI) positron emission tomography/computed tomography (PET/CT) has entered the clinical arena as a novel, non-invasive imaging biomarker for malignant neoplasms [9]. As an underlying rationale on a cellular level, cancer-associated fibroblasts are crucially involved in tumorigenesis and thus, this radiotracer may even serve as a potential substitute for F-18 fluorodeoxyglucose (F-18 FDG) PET/CT in varying oncological scenarios [10, 11]. Of note, in those cancer cohorts, incidental joint uptake on FAPI-directed PET/CT in the shoulders and in patients with rheumatoid arthritis has already been described [12–15]. Expression of FAP in fibroblasts is required for the transient regenerative phase of normal tissue reconstruction, but is overexpressed in chronic pathological processes, causing fibrosis in the tissue [16]. In IgG_4_-related diseases, F-18 FDG, which indicates the degree of glycolysis, can be used as an indicator of inflammation, and fibroblast-targeting radiotracers as a potential biomarker of fibrosis [17]. Thus, we aimed to provide a comprehensive characterization of FAPI upregulation in shoulder joints, thereby determining its role as a non-invasive image biomarker of chronic fibrosis and provide a head-to-head comparison with the inflammation-targeting reference F-18 FDG.

## Materials and Methods

### Patients

In this retrospective study, we analyzed patients with solid malignant tumors who underwent Ga-68 FAPI-04 PET/computed tomography (CT). All patients gave their written informed consent to undergo imaging. The local institutional review board of University Hospital Würzburg waived the need for approval (No. 20210415 02). Patients with sites of metastatic disease in the shoulders (or in surrounding tissue) were not considered for the present analysis. Parts of this cohort have been investigated previously [18–24], without assessing radiotracer accumulation in shoulder joints. Prior to molecular imaging, 52/77 (67.5%) did not receive any treatment, while in the remaining 25/77 (32.5%) anti-tumor therapy had been initiated (surgery in 23/25 [90%], chemotherapy in 17/25 [68%], and prior radiation therapy in 7/25 [28%]). Median time frame between treatment and PET/CT was 74 days. All methods were carried out in accordance with relevant guidelines and regulations. Information on shoulder pain or known joint disease (e.g., rheumatic disease, trauma, and handedness) were not available due to the retrospective character of this study.

### PET/CT Imaging

F-18 FDG PET/CT was conducted after or prior to Ga-68 FAPI-04 PET/CT, without administering any anti-inflammatory drugs between both examinations. None of the patients had a known history of inflammatory diseases. Ga-68 FAPI-04 (average 137 ± 26 MBq) was injected intravenously without fasting. F-18 FDG (3.7 MBq/kg body weight) was also administered intravenously, but a fasting protocol was applied (minimum 6 h prior to radiotracer i.v.). One hour post-injection of either Ga-68 FAPI-04 or F-18 FDG, PET imaging from the vertex of the scull to the proximal thighs with 2-min emission scan per bed position was performed using a Biograph mCT 64 or 128 (Siemens Healthineers, Erlangen, Germany). All patients lifted both arms during the scans. PET images were reconstructed in a 200 × 200 matrix with 4.0 × 4.0 × 5.0 mm voxel size using three-dimensional ordered subset expectation maximization (3D-OSEM) with 3 iterations and 24 subsets, along with Gaussian filter of 2-mm full width at half maximum. Attenuation correction was also performed using transmission CT. We applied the following parameters: matrix, 512 × 512; slice thickness, 5 mm; tube voltage 120 keV; automated tube current modulation (CARD Dose4D, quality reference of 80—120 mAs); tube rotation time, 0.5 s; increment, 30 mm/s; and pitch index of 1.4 [23].

### Visual Assessment

Two experienced nuclear physicians (YM and TH) interpreted all Ga-68 FAPI-04 PET/CT images using a dedicated viewing software in a consensus setting. Although the shoulder is composed of five joints [25], we investigated radiotracer accumulation in the acromioclavicular (AC) joint or the glenohumeral plus subacromial (GH + SA) joints (or both, i.e., AC in combination with GH + SA). On a visual assessment, uptake in those joints can be easily isolated from the body trunk on anterior–posterior maximum intensity projections (MIP) images of PET. Due to their close anatomical localization, we decided to evaluate GH and SA joints together. The sternoclavicular joint was omitted due to difficulties to accurately assess uptake of the joint on anterior–posterior MIP images because the uptake was in close proximity to or covered by lesions in other organs such as the spine, thyroid, lungs, and lymph nodes. Two readers determined whether the joints had more intense uptake than the surrounding structures. The patients were also classified according to whether the patients had none, unilateral, or bilateral FAPI-positive shoulder(s) to investigate their age-related influence on FAPI accumulation.

### Quantitative Analysis

**Fig. 1:**
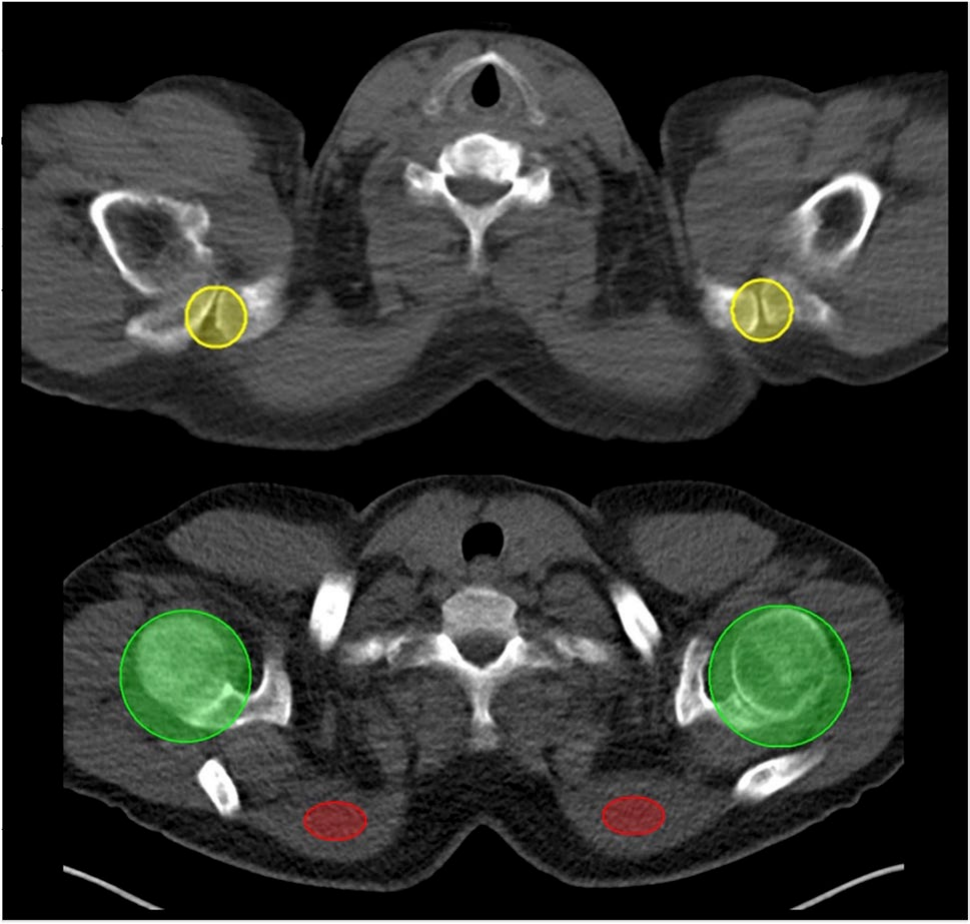
Example of volumes of interest (VOIs) in the shoulder joints. Yellow, green, and red ellipsoids indicate VOIs of the acromioclavicular (AC), the glenohumeral plus subacromial (GH + SA) joints, and the trapezius muscles, respectively

Image analysis was conducted using a syngo.via workstation (VB50; Siemens Healthcare Erlangen, Germany). Volumes of interest (VOIs) were placed by using transaxial CT images as anatomical guidance. Respective ellipsoid VOIs provided maximum standardized uptake values (SUV) and were placed on the bilateral AC joints and shoulder joints. Overlap was carefully avoided (Fig. 1). We also determined target-to-background (TBR) ratios with uptake derived from the trapezius muscles serving as reference. TBR was then calculated as follows: Joints (maximum SUV) / Muscle (mean SUV).

### Statistical Analysis

Statistical analysis was performed using GraphPad Prism 9.0 (GraphPad Software, Inc., San Diego, CA, USA). Quantitative data were expressed as mean ± standard deviation (SD). The difference in age between groups were tested using one-way analysis of variance (ANOVA). The difference in SUV between groups were tested using a Student *t* test. Correlation coefficients were calculated with Pearson’s correlation analysis. A *P* value of less than 0.05 was considered statistically significant.

## Results

### Clinical Characteristics

Among 77 patients (28 females; mean age, 63.1 ± 11.6 years), diagnoses were as follows: cervical cancer (*n* = 25), neuroendocrine tumor (*n* = 19), lung cancer (*n* = 10), pancreatic cancer (*n* = 7), hepatocellular carcinoma (*n* = 6), cancer of unknown primary (*n* = 3), esophageal cancer (*n* = 1), gastric cancer (*n* = 1), breast cancer (*n* = 1), colon cancer (*n* = 1), gastrointestinal stromal tumor (*n* = 1), sarcoma (*n* = 1), and osteomalacia (*n* = 1). A total of 154 shoulders was analyzed. 54/77 (70.1%) patients also underwent F-18 FDG PET/CT within 2.6 ± 9.4 days after or prior to Ga-68 FAPI-04 PET/CTs.

### Visual Assessment Revealed Increased FAP Uptake in Almost Half of the Investigated Shoulders

**Fig. 2:**
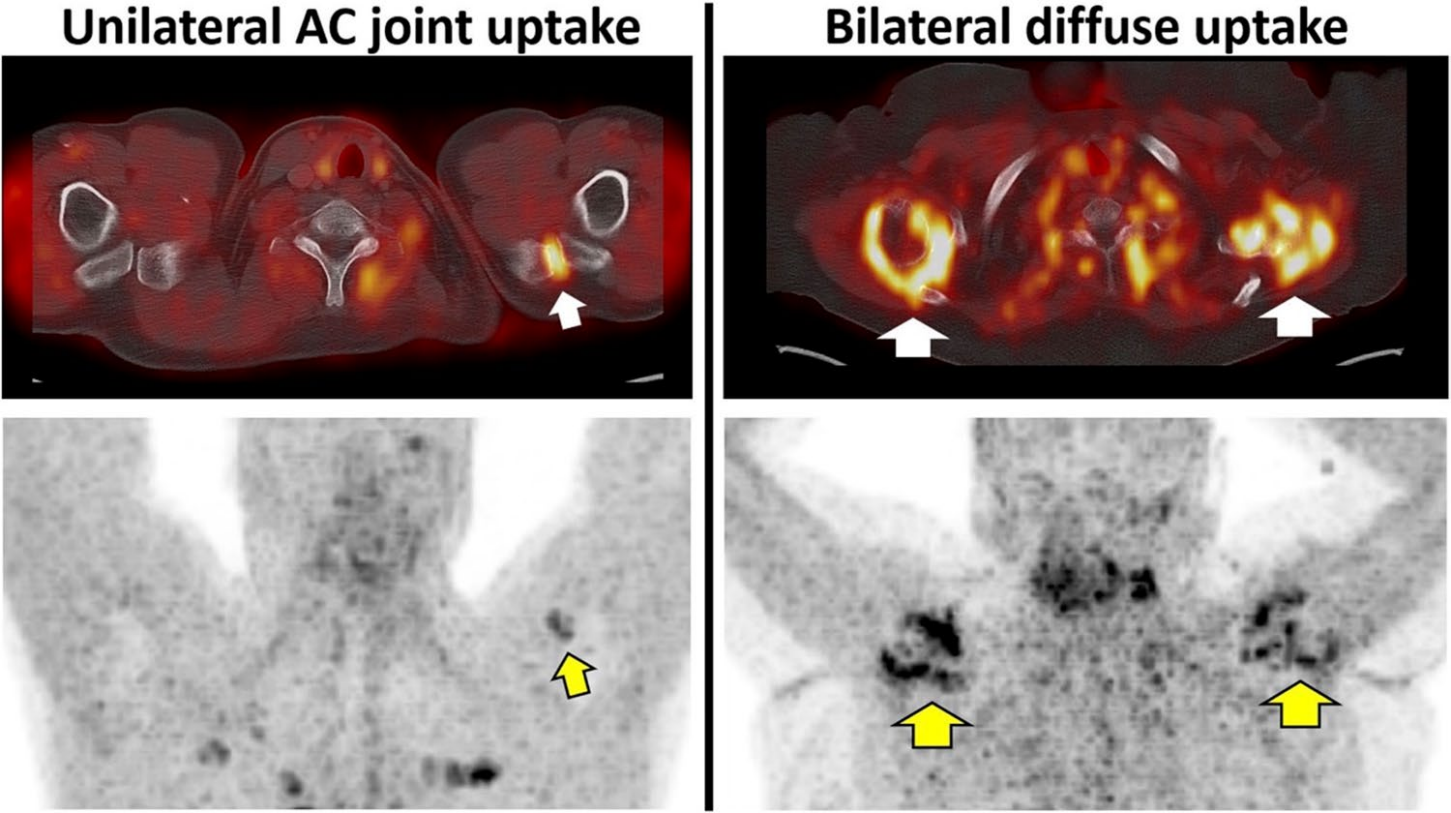
Representative images of Ga-68 FAPI-04 PET. Upper, PET/CT fused images; Lower, PET maximum intensity projection (MIP) images. Arrows indicate intense FAPI uptake in the shoulders. Left, unilateral acromioclavicular joint (AC) uptake of 37-year-old man; Right, bilateral diffuse uptake including uptake in the AC and the glenohumeral plus subacromial joints of 54-year-old woman

Among the 154 shoulders analyzed, 67 shoulders (43.5%) demonstrated increased Ga-68 FAPI-04 accumulation in the shoulder joints, which were distributed as follows: AC joints in 25/67 (37.3%; Fig. 2), followed by GH + SA joints in 23/67 (34.3%), or both (AC and GH + SA joints) in the remaining 19/67 (28.4%; Fig. 2). The difference of age among the patients with none, unilateral, or bilateral shoulder(s) with increased FAPI expression was not significant (61.8 ± 11.4, *n* = 32; 61.5 ± 11.5, n = 28; 68.4 ± 11.3, *n* = 17; *p* = 0.11).

### *In-vivo* FAP Expression was Significantly Elevated When Compared to F-18 FDG

In 108 shoulder joints (54 patients) with available F-18 FDG PET/CTs, 49 shoulders (45.4%) demonstrated visually increased uptake, which was mainly located in AC joints in 20/49 (40.8%), followed by GH + SA joints in 18/49 (36.7%), or both (AC and GH + SA joints) in the remaining 11/49 (22.4%).

**Fig. 3:**
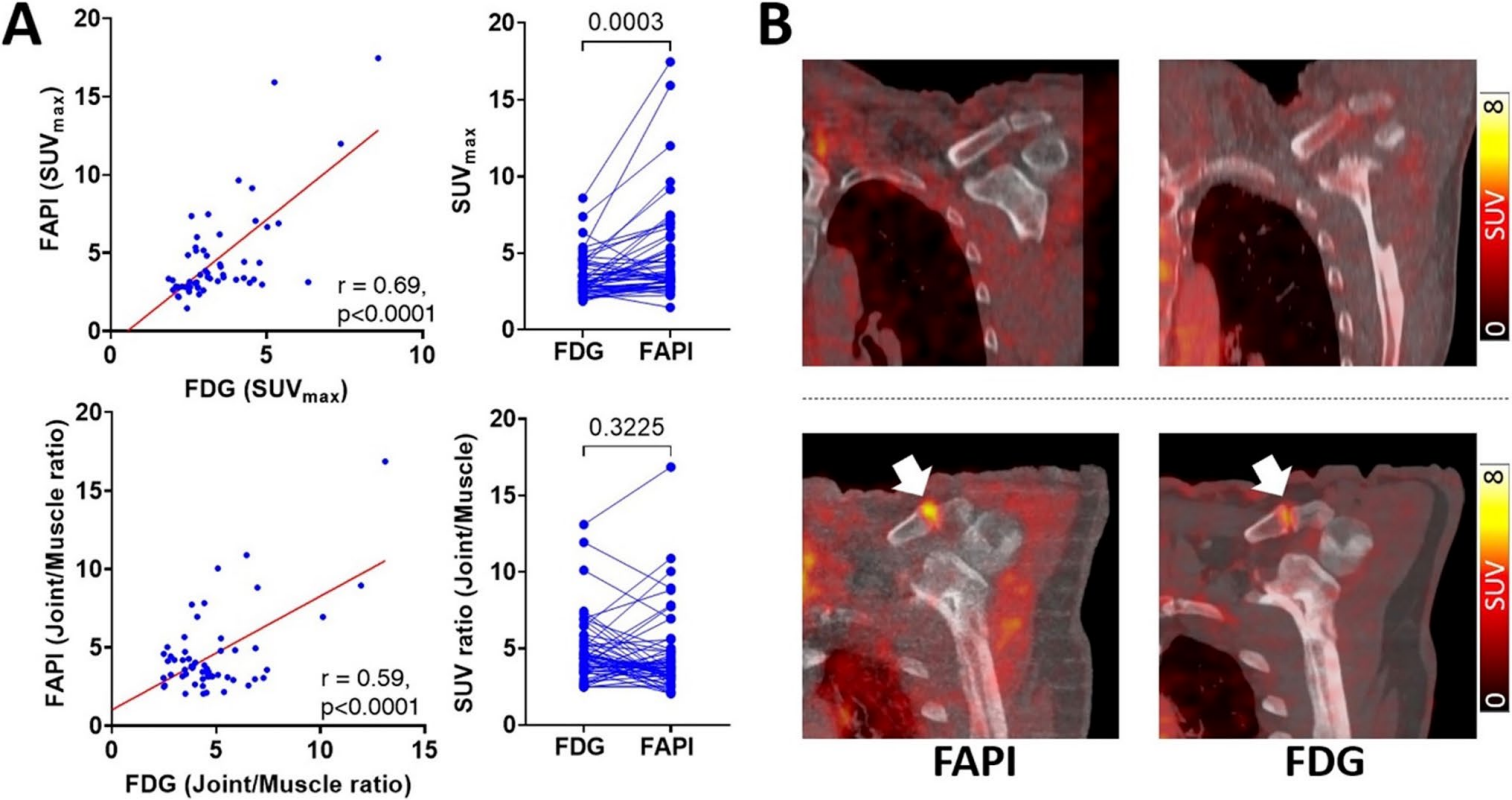
Comparison of Ga-68 FAPI-04 (FAPI) and F-18 FDG (FDG) accumulation. FAPI-derived maximum standardized uptake values (SUV_max_) were well correlated with SUV_max_ obtained from FDG (A, upper, left), while FAPI SUV_max_ in the shoulders was significantly increased (A, upper, right). FAPI-derived target-to-background ratios (TBRs) were also well correlated with TBR of FDG (A, lower, left), while TBR of FAP uptake in the shoulders was not significantly increased, but also demonstrated a trend towards higher values on FAPI-directed imaging (**A**, lower, right). Representative examples with no focal uptake on both FAPI and FDG PET/CTs (upper rows) in 52-year-old man and uptake-positive shoulders (in acromioclavicular joints) on both FAPI and FDG PET/CTs (lower rows) in 78-year-old man (**B**)

Among the 108 shoulder joints in a visual read, 56 joints demonstrated increased uptake on both imaging modalities. Comparing both signals, 28/56 (50.0%) shoulder joints had stronger uptake on Ga-68 FAPI-04 PET than that on F-18 FDG PET; 15/56 (26.8%) joints had equivalent uptake; and only 13/56 (23.2%) joints exhibited less *in-vivo* FAP expression. On a quantitative assessment, SUVs derived from both radiotracers were well correlated (*r* = 0.69, *p* < 0.0001), while *in-vivo* FAP expression was significantly higher relative to F-18 FDG (4.7 ± 3.2 vs 3.6 ± 1.3, *p* < 0.001; Fig. 3).

Moreover, we observed no significant differences between TBR of F-18 FDG and Ga-68 FAPI-04 (F-18 FDG, 4.8 ± 2.1 vs. Ga-68 FAPI-04, 4.5 ± 2.6, *p* = 0.32). Comparable to SUV, significant correlation between TBR of both radiotracers was recorded (*r* = 0.59, *p* < 0.0001).

## Discussion

In a large cohort of cancer patients imaged with Ga-68 FAPI-04 PET/CT, we observed visual increased FAPI expression in more than 43% of patients, while distribution was relatively balanced among investigated AC or GH + SA joints (or both). In addition, frequency and *in-vivo* FAP expression was also markedly elevated when compared to the reference radiotracer F-18 FDG. There are various types of PET and SPECT tracers that visualize inflammation, e.g. targeting C-X-C motif chemokine receptor 4 or somatostatin receptor [26, 27]. FAPI-directed radiotracers, however, are directed towards fibroblasts, which are responsible for joint stiffness and contractures [5] and thus, *in-vivo* assessment of fibroblast activity could be related not only to pain, but also to restricted joint mobility function. Thus, Ga-68 FAPI-04 PET may provide a novel image biomarker to assess chronic remodeling in shoulder joints *in-vivo*. The herein presented findings may then enable for rigorous assessments in patients affected with RA or OA, e.g., to initiate FAP-tPDT for synovial depletion based on FAPI PET signal strength. Such an image-guided photodynamic treatment would then allow to adjust light dose for providing improved therapeutic efficacy while identifying peri-arthritic unaffected tissue prior to treatment on-set, ultimately avoiding off-target effects. Further indications include longitudinal response monitoring, e.g., to determine response based on declining FAPI PET signal after FAP-tPDT.

In our study, Ga-68 FAPI-04 uptake in the AC joints was often visible as focal hot spots on MIP images, thereby allowing a reliable visual read-out of affected joints. Although smaller than GH + SA joints, AC joints were also more often FAP + . AC joint arthritis is common and it often accompanied by other shoulder conditions such as rotator cuff disease or glenohumeral arthritis [28]. Of note, AC joint-related pathology is also frequently observed in relatively young patients, including over 30 years of age [29]. Based on our findings, however, shoulder uptake in AC joints is relatively common and thus, the interpreting nuclear medicine expert should be aware of such a potential pitfall in scan interpretation, e.g., when investigating a FAP + (metastatic) lymph node in close proximity to the herein investigated anatomical localizations. In this regard, a careful assessment of MIP images can already provide a useful hint whether respective joints are affected, while transaxial PET/CT reads can then rule out (or corroborate) uptake in AC joints (Fig. 2). In addition, recently introduced standardized reporting systems for theranostic radiotracers may also be useful to segregate between benign and malignant findings [30], once such frameworks become available in the context of FAPI-directed molecular imaging.

Clinical implication of Ga-68 FAPI-04 uptake in shoulder joints have not been fully elucidated yet. Since shoulder joint diseases include frozen shoulder, OA, RA and rotator cuff tear [31], FAPI uptake may be most likely associated with or caused by such pathological conditions. In the present study, the difference of age among the patients with none, unilateral, or bilateral shoulder(s) with increased FAPI expression was not significant. However, the present study included patients with malignancies in particular in elder subjects (88% of patients were aged 50 years or older). We cannot conclude that age does not correlate with quantified Ga-68 FAPI-04 joint uptake, because a relevant portion of patients is affected with shoulder diseases already in their 40 s and 50 s [29]. Thus, further studies including a broader age spectrum along with a rigorous assessment of shoulder pain prior to scanning are needed to link the herein derived uptake pattern and signal strength to age-related (chronic) shoulder pain. Nonetheless, frozen shoulder, RA and OA are all associated with fibroblast-mediated, chronic inflammation [32, 33]. For instance, FAP deficiency in dedicated mouse models led to less cartilage degradation [34]. Of note, a recent *ex-vivo* study investigating RA synovial explants demonstrated that anti-FAP targeted photodynamic therapy (FAP-tPDT) caused cell death on *ex-vivo* activated synovial fibroblasts with increasing light dose [35]. This FAP-tPDT may enable for rigorous assessments in patients affected with RA or OA, e.g., to initiate FAP-tPDT for synovial depletion based on FAPI PET signal strength. Such an image-guided photodynamic treatment would then allow to adjust light dose for providing improved therapeutic efficacy while identifying peri-arthritic unaffected tissue prior to treatment on-set, ultimately avoiding off-target effects. Moreover, Wandler and coworkers used dedicated questionnaires and investigated whether F-18 FDG may be a useful tool to segregate between various joint inflammation or injuries (with OA and bursitis being associated with diffuse uptake pattern) [36]. While F-18 FDG PET/CT may emerge as a relevant radiotracer in an active inflammatory setting right after disease on-set or shoulder damage [36], FAPI-targeted PET has been advocated to play a role in chronic destructive processes and thus, future studies may then also conduct such pain-related, patient-centered registries in the context of FAPI-PET/CT [37]. Of note, those considerations are further fueled by the fact that a recent trial investigating uptake on FAPI PET/CT in benign diseases also reported on the spine, followed by the shoulders as the most frequent locations of relevant *in-vivo* FAP upregulation in patients with arthritis or inflammatory-related abnormalities [13].

This study has several limitations. As one of the most complex joints in the human body including the rotator cuff, bursa, and ligaments, reference CT images may not allow for such a detailed anatomical assessment to reliably segregate PET uptake in all of those localizations. PET combined with MRI may overcome this issue [13]. Since it was a retrospective analysis, information on the patient's symptoms, formal OA/RA diagnosis and treatments affecting shoulder uptake, such as anti-inflammatory drugs, were not available. The data of the body height for body mass index, smoking history, blood pressure, and blood tests were also not available for this study. Future studies may also conduct matched-pair comparisons, e.g., in patients with and without symptoms and more rigorous use of joint-focused X-rays and/or MRI [38, 39]. Long-term evaluation is also needed to clarify the relationship between Ga-68 FAPI-04 uptake signal and disease progression, preferably by including a larger number of patients in a prospective study.

## Conclusions

We systemically characterized Ga-68 FAPI-04 uptake in shoulder joints and observed relevant radiotracer accumulation in more than 43% of the subjects, in particular in the AC, followed by GH + SA joints in oncological studies. Quantified FAP expression demonstrated correlation with F-18 FDG uptake, but *in-vivo* FAP expression was significantly elevated, thereby indicating that Ga-68 FAPI-04 signal strength may provide information on chronic remodeling rather than active inflammation-related joint destruction. The herein presented findings may trigger future studies for image-piloted strategies in patients with OA or RA, e.g., to initiate or adjust FAPI-targeted, reparative interventions at the time point of the maximum of target expression, including photodynamic therapies.

## Data Availability

The datasets used and/or analysed during the current study available from the corresponding author on reasonable request.
